# Design, synthesis and investigation of water-soluble hemi-indigo photoswitches for bioapplications

**DOI:** 10.3762/bjoc.15.275

**Published:** 2019-11-22

**Authors:** Daria V Berdnikova

**Affiliations:** 1 Department Chemie–Biologie, Organische Chemie II, Universität Siegen, Adolf-Reichwein-Str. 2, 57076 Siegen, Germany

**Keywords:** hemi-indigo, molecular switches, photochromism, photoswitching, visible light, water solubility

## Abstract

A series of hemi-indigo derivatives was synthesized and their photoswitching properties in aqueous medium were studied. The dimethoxy hemi-indigo derivative with the best photochromic performance in water was identified as a promising platform for the development of photoswitchable binders for biomolecules. The synthetic approach towards the introduction of the alkylamino pendant to the dimethoxy hemi-indigo core was developed that allowed to obtain an RNA-binding hemi-indigo derivative with photoswitchable fluorescent properties.

## Introduction

The application of organic photochromes in biological systems is fraught with their poor solubility and reduced photoswitching efficiency in aqueous medium. In many cases, approaches to improve the water solubility by chemical modification of the photochromic scaffolds are not straightforward because the introduction of substituents often interferes with the desired photochemical properties. Along these lines, special efforts have been devoted to design, for example, water-soluble derivatives of spiropyran [1–3], azobenzene [4–5], diarylethene [6–8], or chromene [9] that keep efficient photochromism in aqueous medium. Although a significant progress has been made in the development of water-soluble photochromes, there is still an emerging search for new types of photochromic compounds for applications in biological systems. In particular, nowadays the development of photopharmacology is based mainly on azobenzene chemistry [10–11] and, therefore, finding of new biocompatible photochromes with complementary properties is highly desirable to speed up the progress in this important field. In this context, an emerging class of hemi-indigo photoswitches attracted special attention [12–17]. Despite the fact that the hemi-indigo dyes are known for more than 100 years [18], their photochemical properties are still underexplored and no targeted studies on their photoswitching in aqueous media were performed. The hemi-indigo scaffold exists in two forms that can be photoswitched reveresibly. In most cases, the *Z*-isomer of hemi-indigo is thermodynamically stable whereas the *E*-isomer is metastable. However, if the indoxyl nitrogen is substituted with alkyl or aryl groups, both isomers of hemi-indigo have very close energies and none of them is clearly preferred thermodynamically [13]. The absorption spectrum of the *E*-isomer is bathochromically shifted relative to the one of the *Z*-isomer. Depending on the substitution pattern, the thermal lifetimes of metastable *E*-isomers at 25 °C vary from hours to days and sometimes even years [12–14]. Remarkably, for sterically hindered hemi-indigo derivatives, thermal lifetimes of the metastable states beyond 3000 years have been achieved [14]. Unlike most of the widely applied photochromes (spiropyrans, spirooxazines, chromenes, dithienylethenes, etc.), both forms of hemi-indigo absorb in the visible light region. Therefore, photochemical switching does not require the use of the UV light, which is of high importance for biological applications.

Herein, the synthesis and adjustment of the substitution pattern of hemi-indigo derivatives for the efficient photoswitching in aqueous medium are described. Detailed characterization of the photoinduced isomerization of hemi-indigo derivatives in water is provided. Additionally, synthetic peculiarities of the introduction of an RNA-affine alkylamino substituent to the hemi-indigo scaffold are discussed.

## Results and Discussion

### Synthesis of hemi-indigo derivatives *Z*-**1a–c**

**Scheme 1:**
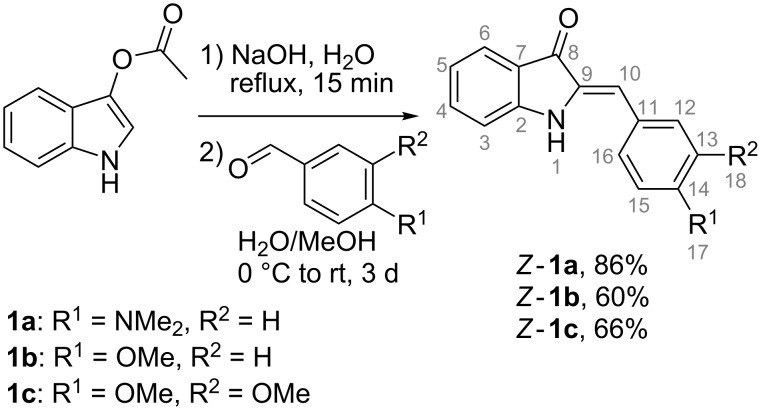
Synthesis of hemi-indigo derivatives *Z*-**1a**–**c**.

The synthesis of hemi-indigo derivatives *Z*-**1a**–**c** with different substitution patterns of the phenyl ring was performed through the aldol condensation of indoxyl-3-acetate with the corresponding benzaldehydes under alkaline conditions (Scheme 1) [13]. All compounds **1a**–**c** were obtained in good yields as pure *Z*-isomers as supported by the NMR data (Figures S5–S13 in Supporting Information File 1).

### Introduction of an alkylamino substituent to the hemi-indigo scaffold

**Scheme 2:**
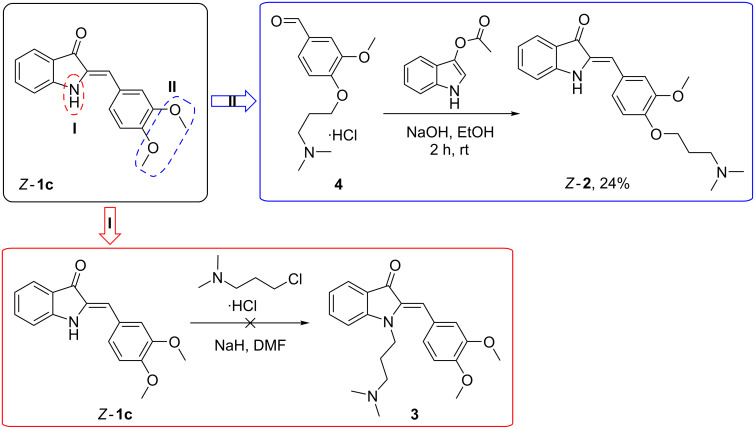
Synthetic routes to alkylamino-substituted dimethoxy hemi-indigo *Z*-**1c**.

Based on the data on photoswitching in water (vide supra), the dimethoxy-substituted hemi-indigo *Z*-**1c** was selected as a core structure for the design of RNA binders with photoswitchable properties [12]. To increase the solubility in aqueous medium and potential RNA-binding properties, the dimethylaminopropyl substituent [19] was introduced to hemi-indigo *Z-***1c**. Two synthetically straightforward approaches of the alkylamino group introduction were considered: (i) *N*-alkylation of the indoxyl core and (ii) *O*-alkylation of the dimethoxyphenyl residue (Scheme 2).

Surprisingly, compound **3** (method I) could not be obtained due to the cleavage of the C–C double bond in the course of the reaction followed by extensive destruction of the heterocyclic fragment. Variation of the reaction conditions, e.g., reduction of the reaction temperature and time, changing the ratio of the reactants and addition of NaI as a catalyst, did not suppress this decomposition to a significant extent. A possible reason for this side process is the reactivity of the double bond carbon atom (Michael acceptor) [20]. The intramolecular nucleophilic attack of the introduced alkylamino group can possibly lead to the immediate double-bond cleavage in **3**. This results in formation of unstable indoxyl that undergoes further destruction and veratraldehyde that is detected in the reaction mixture by NMR. This assumption is supported by the observation that only one hemi-indigo derivative bearing an alkylamino substituent of a shorter length on the indoxyl N atom has been reported so far [20].

Modification of the phenyl ring by method II was successful and allowed to obtain the desired hemi-indigo **2** as a pure *Z*-isomer with 24% yield (Scheme 2) [12]. Importantly, the synthesis of *Z-***2** required milder conditions and shorter reaction times than that of derivatives *Z*-**1a**–**c**. Thus, the use of the water/methanol mixture as a solvent, heating and extended reaction times of more than 2 h resulted in the destruction of the desired product *Z-***2**. At the same time, performing the reaction at room temperature in pure ethanol allowed to increase the yield of *Z-***2** and to reduce the number of side-products. Purification of hemi-indigo *Z-***2** by conventional column chromatography was not efficient. However, pure product *Z-***2** could be obtained by gel filtration chromatography on sephadex (MeOH); the isolated compound *Z*-**2** is stable in its free base form whereas its hydrochloride salt slowly decomposes.

### Optical properties and photoswitching in aqueous medium

**Figure 1:**
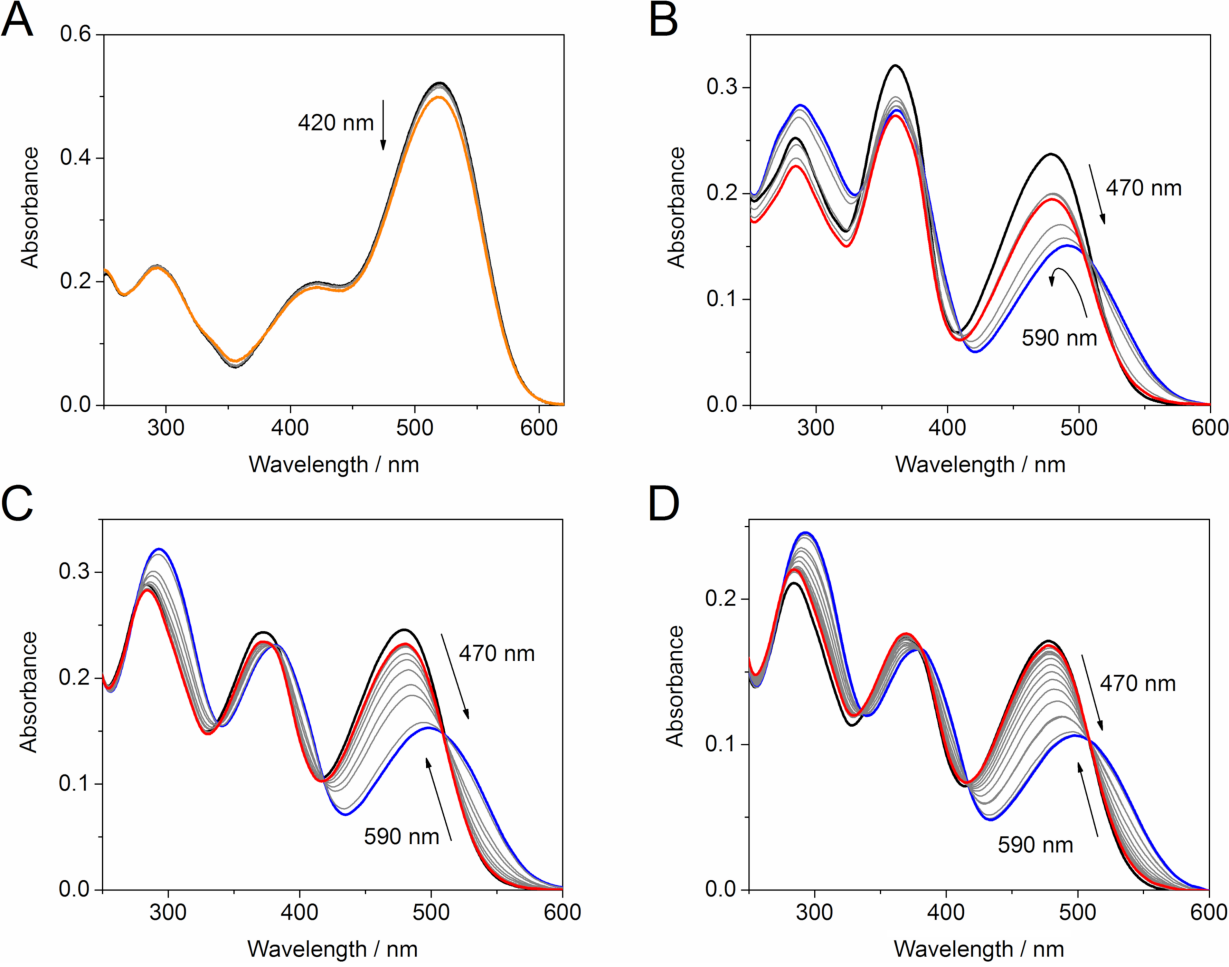
Photoswitching of hemi-indigo derivatives: (A) *Z*-**1a**, *c* = 20 μM in H_2_O with 10% (v/v) DMSO, λ_ex_ = 420 nm; (B) *Z*-**1b**, *c* = 20 μM in H_2_O with 2% (v/v) EtOH, λ_ex_ = 470 nm (forward reaction) and 590 nm (backward reaction); (C) *Z*-**1c**, *c* = 20 μM in H_2_O with 2% (v/v) EtOH, λ_ex_ = 470 nm (forward reaction) and 590 nm (backward reaction); (D) *Z*-**2**, *c* = 15 μM in H_2_O, λ_ex_ = 470 nm (forward reaction) and 590 nm (backward reaction), 20 °C. Spectra of the initial *Z*-isomers: black; PSS^420^: orange; PSS^470^: blue; PSS^590^: red.

Hemi-indigo derivatives *Z*-**1a**–**c** display intense long-wavelength absorption bands, whose maxima are clearly dependent on the strength of the electron-donating substituent in the 4-position of the phenyl ring (Figure 1, Table 1). Thus, the exchange of the 4-dimethylamino group in *Z*-**1a** for a 4-methoxy group in *Z*-**1b** resulted in a significant blue shift of the absorption maximum (Δλ = 42 nm). Notably, the introduction of a second methoxy group to the 3-position of the phenyl ring in *Z*-**1c** did not shift the absorption maximum and just slightly affected the extinction (Table 1). Hemi-indigo derivatives *Z*-**1a**–**c** are not fluorescent in aqueous solution.

**Table 1:** Photochemical and photophysical properties of hemi-indigo derivatives **1a**–**c** and **2** in water.

Species	λ_abs_ (*Z*)/nm	ε (*Z*)/ L mol^−1^ cm^−1^	λ_abs_ (*E*)/ nm^a^	ε (*E*)/ L mol^−1^ cm^−1a^	PSS^470^/ (% *Z*/*E*)^a^	PSS^590^/ (% *Z*/*E*)^a^	Φ_Z-E_/Φ_E-Z_ / 10^−2 b^	ΔG*/ kcal mol^−1 c^	*t*_1/2_ 25 °С^d^

**1a**^e^	521	26039	n.d.^f^	n.d.^f^	n.d.^f^	n.d.^f^	n.d.^f^	n.d.^f^	n.d.^f^
**1b**	479	11922	508	6727	28/72	n.d.^g^	3.8/n.d.^g^	26.5	47 d
**1c**	479	12086	515	7127	21/79	93/7	2.6/0.2	26.2	31 d
**2**	478	10549	515	6368	24/76	97/3	2.4/0.1	26.1	25 d
**2**^h^	478	10456	514	6289	20/80	97/3	2.7/0.2	23.7	11 h

^a^Calculated according to Fischer [21]. ^b^Photoisomerization quantum yields of the forward Φ_Z-E_ (at 470 nm) and backward Φ_E-Z_ (at 590 nm) reactions. ^c^Free activation enthalpies for the thermal *E–Z* isomerization. ^d^Half-lifes of the *E*-form at 25 °C. ^e^In water containing 10% (v/v) DMSO. ^f^Compound shows very weak photochromism in aqueous medium. ^g^The backward switching was not complete due to precipitation of the compound after 2 h of irradiation. ^h^The data were obtained in aqueous 10 mM Na-phosphate buffer containing 0.1 M NaCl, pH 7.0 [12].

**Scheme 3:**
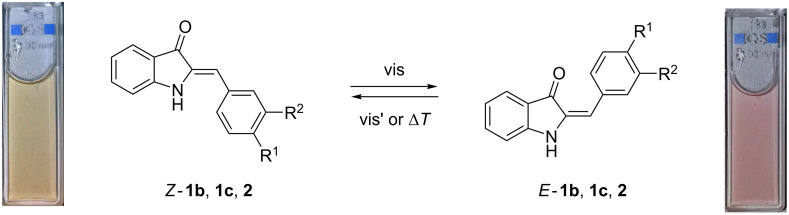
Photoswitching of hemi-indigo derivatives.

To assess the potential applicability of hemi-indigo derivatives *Z*-**1a**–**c** in biological systems, photoswitching of these compounds was tested in aqueous medium (Scheme 3). The forward reaction was performed upon irradiation at 360 (UV), 420 nm (violet), 470 nm (blue) and 520 nm (green) light (Figure 1 and Figures S1 and S2 in Supporting Information File 1).

Surprisingly, almost no switching of the dimethylamino-substituted hemi-indigo *Z*-**1a** was observed in water with 10% DMSO, i.e., only irradiation with violet light (420 nm) led to the residual isomerization (Figure 1A). Additionally, the hemi-indigo *Z*-**1a** was hardly soluble in aqueous medium and rather fast precipitation took place even in the presence of the co-solvent. The comparison with the reported data on *Z*-**1a** [13] and related hemi-indigo derivatives containing a 4-amino group in the phenyl ring [13] allowed to conclude that this substitution pattern is unfavorable for photoswitching in aqueous medium. Thus, a higher content of organic co-solvents (20–30% of DMSO, DMF or THF) or/and the presence of triethylamine was required to stimulate the photoswitching of the reported compounds with a 4-amino group in the phenyl ring [13]. Considering the limitations imposed on the nature and content of organic co-solvents used in biological studies, the dimethylamino derivative *Z*-**1a** was excluded from further studies.

**Figure 2:**
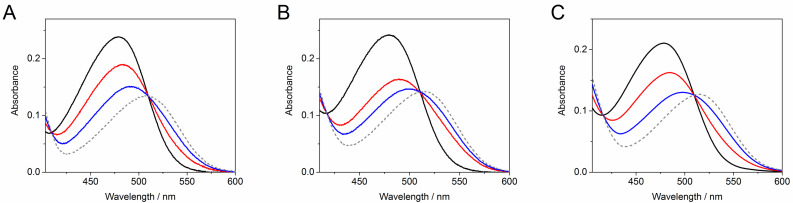
Absorption spectra of the *Z*-isomer (black), two photostationary states obtained upon irradiation with 520 nm (red) and 470 nm (blue) light for (A) **1b**, (B) **1c**, (C) **2** in water, *c* = 20 μM, 20 °C. Grey dashed line represents the spectra of the corresponding *E*-isomers calculated by the Fischer method.

In contrast, mono- and dimethoxy-substituted hemi-indigo derivatives *Z*-**1b** and *Z*-**1c** showed pronounced spectral changes upon irradiation indicating an efficient *Z*–*E* isomerization of the C–C-double bond (Figure 1B and Figure 1C). The most complete *Z*–*E* conversion for both compounds *Z*-**1b** and *Z*-**1c** was achieved upon irradiation with blue light (470 nm) (cf. Figures S1 and S2, Supporting Information File 1). The photoreactions proceeded rather fast and the photostationary state PSS^470^ was reached within 2.5 min for compound *Z*-**1b** and within 3.0 min for compound *Z*-**1c**. The backward *E*–*Z* conversion from PSS^470^ was performed by irradiation with 590 nm (amber) light and occurred much slower (Table 1). In the case of monomethoxy derivative *E*-**1b**, the backward reaction from PSS^470^ proceeded successfully during ca. 2 h of irradiation after which the isosbestic point was lost and the absorption intensity started decreasing due to slow precipitation of the compound from the aqueous solution (Figure 1B). The presence of the second methoxy group ensured a better stability of aqueous solutions of the dimethoxy derivative *Z*-**1c**. In this case, the backward *E*–*Z* isomerization of *E*-**1c** from PSS^470^ took place within 5 h resulting in almost complete restoration of the initial absorbance of *Z*-**1c** in the PSS^590^ (Figure 1C). Investigation of the photostationary mixtures by fluorescence spectroscopy revealed that the photoinduced isomers *E*-**1b** and *E*-**1c** are not fluorescent in aqueous medium. The analysis of the isomeric compositions of the photostationary states was performed by the Fischer method [21] because NMR-spectroscopic analysis was precluded by insufficient solubility or/and possible aggregation at higher concentrations. Thus, the Fischer method allowed to calculate the absorption spectra of pure *E*-isomers of **1b** and **1c** in water (Figure 2) as well as to evaluate the extent of the *Z*–*E* conversion in PSS^470^ and PSS^590^ (Table 1).

The dimethoxy derivative *Z*-**1c** showed better *Z–E* conversion in PSS^470^ and a larger difference between the absorption maxima of the *Z-* and *E*-forms. At the same time, the introduction of the second methoxy group to the phenyl ring resulted in a decrease of the *Z*–*E* isomerization quantum yield and reduced the thermal half-life of *E*-**1c** in comparison to *E*-**1b** (Table 1). Nevertheless, the dimethoxy-substituted hemi-indigo *Z*-**1c** was selected as a core structure for the design of photoswitchable RNA binders due to its higher conversion and better solubility in water.

The introduction of the alkylamino group provided compound *Z*-**2** with much better solubility in water. At the same time, the presence of the alkylamino substituent only slightly influenced the photochemical and photophysical characteristics of the hemi-indigo *Z*-**2** in comparison with the parent compound *Z*-**1c** (Figure 1D, Figure 2C, Table 1). Thus, the positions of the absorption maxima of both, the *Z-* and *E*-forms, the extent of conversion in PSSs, the photoisomerization quantum yields as well as the half-lifes of the photoinduced forms appeared to be almost independent of the presence of the alkylamino substituent. Interestingly, a comparison with the data obtained for *Z*-**2** in aqueous 10 mM Na-phosphate buffer containing 0.1 M NaCl, pH 7.0 [12], showed that the increase in the ionic strength of the medium resulted in a drastic decrease of the half-life of the photoinduced form *E*-**2** whereas other characteristics remained almost unaffected (Table 1). This indicates that the highly ionic medium reduces the energy barrier in the ground state that is responsible for the rate of thermal *E*–*Z* isomerization pointing out the importance of the Coulomb interactions between hemi-indigo and buffer components. A possible explanation of this observation can be provided by the comparison with structurally related hemi(thio)indigo dyes [22]. Thus, in the case of hemi(thio)indigo, the energy maximum in the ground state corresponds to the 90° rotation about the central double bond resulting in formation of a state with biradical-like character that is polarized along the molecule’s long axis [22]. The close structural similarity allows to expect a similar character of the transition state for the hemi-indigo derivatives. Therefore, a highly ionic medium can stabilize the transition state of the hemi-indigo leading to the decrease of the energy barrier between *Z-* and *E*-isomers and, therefore, reducing the half-life of the *E*-form. However, further studies are required to provide detailed explanation of this effect.

Recently, a proof-of-principle for the application of hemi-indigo derivative *Z*-**2** as a binder for the human immunodeficiency virus type 1 (HIV-1) RNA with photoswitchable fluorescent properties was provided [12]. It was shown that hemi-indigo *Z*-**2** associates with the regulatory elements of HIV-1 genome RNA with high affinity (*K*_b_ ≈ 10^5^ M^−1^) while keeping its photoswitching properties. Both, the initial *Z*-**2** and photoinduced *E*-**2** forms remain bound to RNA. Most notably, the interaction of *Z*-**2** with HIV-1 RNA produces a remarkable light-up effect whose extent depends on the particular sequence of RNA. Photoswitching of the RNA-bound hemi-indigo *Z*-**2** to the *E*-form results in emission quenching. The ON–OFF fluorescence switching of *Z*-**2**–RNA complexes can be performed reversibly by repeated irradiation with blue (470 nm) and amber (590 nm) light.

## Conclusion

To sum up, hemi-indigo derivatives with different substitution patterns in the phenyl ring were synthesized and their photochemical behavior in aqueous medium was studied. The presence of a methoxy group in the 4-position of the phenyl ring was identified as a necessary condition for the efficient photoswitching of hemi-indigo in water. At the same time, the presence of a strong electron-donating dimethylamino group at this position is unfavorable for the photoswitching in water. It was also shown that the introduction of a second methoxy group in the 3-position of the phenyl ring improves the water solubility of the photoswitch and increases the red shift of the absorption maximum of the *E*-isomer. As a further step, the synthetic approach towards the attachment of the RNA-affine alkylamino substituent was developed. Overall, the hemi-indigo derivatives were introduced as promising photoswitches for aqueous media possessing valuable properties for bioapplications.

## Experimental

### Materials and equipment

Reagents and solvents were obtained from commercial sources (Acros, Merck, Fischer) and used as received. Reactions were monitored on POLYGRAM^®^ SIL G/UV_254_ (Macherey-Nagel) TLC plates with detection by UV light irradiation (254 nm or 366 nm). Column chromatography was performed on Sephadex^®^ columns. ^1^H NMR and ^13^C NMR spectra were recorded on a JEOL ECZ 500 spectrometer at 25 °C using 5 mm tubes. Chemical shifts were determined with accuracy of 0.01 ppm and 0.1 ppm for ^1^H and ^13^C spectra, respectively, and are given relative to the residual signal of the solvent that was used as internal standard (DMSO-*d*_6_: δ_H_ = 2.50 ppm, δ_C_ = 39.5 ppm). Spin–spin coupling constants for the proton spectra were determined with accuracy of 0.2 Hz. The proton NMR signal assignments were performed using COSY and ROESY 2D NMR techniques. The carbon NMR signal assignments were performed by means of HSQC and HMBC 2D NMR techniques. Mass spectra (ESI) were recorded on a Finnigan LCQ Deca mass spectrometer. Elemental analysis was performed with a HEKAtech EUROEA combustion analyser by Mr. Rochus Breuer (Universität Siegen, Organische Chemie I). Melting points were measured with a BÜCHI 545 (BÜCHI, Flawil, CH) melting point apparatus in open capillaries and are uncorrected. Electronic absorption spectra were measured on a Cary 100 Bio two-beam spectrophotometer and a Specord 600 (Analytik Jena AG) diode-array spectrophotometer. Fluorescence spectra were recorded on a Cary Eclipse spectrofluorimeter. Optical spectroscopy measurements were performed in thermostated quartz sample cells of 10 mm pathlength. Preparation and handling of the solutions were carried out under red light. Photochemical reactions were performed using the following LED light sources: LUMOS (360 nm); Roithner H2A1-H420 130 mW (420 nm); Roschwege HighPower-LED Blau (470 nm); Roschwege HighPower-LED Grün (520 nm); Roschwege HighPower-LED Amber (590 nm).

### Synthesis

The synthesis and characterization of hemi-indigo derivative *Z*-**2** are described in detail in [12].

#### General procedure for the synthesis of hemi-indigo derivatives *Z*-**1a–c**

Under argon gas atmosphere, a solution of indoxyl-3-acetate (200 mg, 1.14 mmol) in aqueous NaOH (1.5 M, 6.2 mL, degassed) was heated at 100 °C for ca. 15 min. Then, the mixture was cooled to 0 °C and a solution of the corresponding aldehyde in 1–2 mL MeOH (Ar degassed) was added upon vigorous stirring (for compound *Z*-**1a**: 4-(dimethylamino)benzaldehyde (170 mg, 1.14 mmol); for compound *Z*-**1b**: *p*-anisaldehyde (155 mg, 1.14 mmol); for compound *Z*-**1c**: veratraldehyde (189 mg, 1.14 mmol)). After the addition of the aldehyde, the mixture was warmed to ambient temperature and stirred for 3 days. Then, the mixture was neutralized with 1 M aq HCl and extracted with EtOAc. The combined organic layers were dried with Na_2_SO_4_ and the solvent was removed in vacuo. The obtained solid was redissolved in EtOH and filtered to remove the insoluble precipitate of indigo side-product. After filtration, the solvent was partially removed and the pure *Z*-isomer of the corresponding product was crystallized at −20 °C.

#### (Z)-2-(4-(Dimethylamino)benzylidene)indolin-3-one (Z-**1a**)

Deep violet needles, yield 86% (259 mg, 0.98 mmol); mp 232–234 °С (lit. [13]: 235–236 °С); *R*_f_ 0.67 (hexane/EtOAc 1:1, v/v); ^1^H NMR (500 MHz, DMSO-*d*_6_) δ 3.00 (s, 6H, H-17), 6.64 (s, 1H, H-10), 6.78 (d, *J* = 9.0 Hz, 2H, H-13, H-15), 6.88 (ddd, *J* = 7.8, 7.0, 0.7 Hz, 1H, H-5), 7.14 (d, *J* = 8.1 Hz, 1H, H-3), 7.47 (ddd, *J* = 8.3, 7.1, 1.3 Hz, 1H, H-4), 7.55 (d, *J* = 8.3. Hz, 1H, H-6), 7.61 (d, *J* = 8.8 Hz, 2H, H-12, H-16), 9.55 (s, 1H, H-1) ppm; ^13^C NMR (126 MHz, DMSO-*d*_6_) δ 39.7 (2C, C-17), 112.1 (2C, C-13, C-15), 112.5 (1C, C-3), 112.6 (1C, C-10), 119.1 (1C, C-5), 120.5 (1C, C-7), 121.3 (1C, C-11), 123.7 (1C, C-6), 131.72 (2C, C-12, C-16), 131.68 (1C, C-9), 135.3 (1C, C-4), 150.3 (1C, C-14), 153.3 (1C, C-2), 185.2 (1C, C-8); Anal. calcd for C_17_H_16_N_2_O: C, 77.25; H, 6.10; N, 10.60; found: C, 77.05; H, 6.13; N, 10.39 %; ESIMS (MeOH, *m*/*z*): 263 [*Z*-**1a** − H]^−^, 264 [*Z* − **1a**]^+^, 265 [*Z*-**1a** + H]^+^.

#### (Z)-2-(4-Methoxybenzylidene)indolin-3-one (Z-**1b**)

Brownish-golden powder, yield 60% (172 mg, 0.68 mmol); mp 180–181 °С (lit. [20]: 180–181 °С); *R*_f_ 0.75 (hexane/EtOAc 1:1, v/v); ^1^H NMR (500 MHz, DMSO-*d*_6_) δ 3.82 (s, 3H, H-17), 6.65 (s, 1H, H-10), 6.90 (t, , *J* = 7.8 Hz, 1H, H-5), 7.04 (d, *J* = 8.8 Hz, 2H, H-13, H-15), 7.14 (d, *J* = 8.1 Hz, 1H, H-3), 7.51 (ddd, *J* = 8.4, 7.1, 1.1 Hz, 1H, H-4), 7.57 (d, *J* = 7.6 Hz, 1H, H-6), 7.71 (d, *J* = 8.8 Hz, 2H, H-12, H-16), 9.68 (s, 1H, H-1) ppm; ^13^C NMR (126 MHz, DMSO-*d*_6_) δ 55.3 (1C, C-17), 110.5 (1C, C-10), 112.6 (1C, C-3), 114.6 (2C, C-13, C-15), 119.5 (1C, C-5), 120.2 (1C, C-7), 123.9 (1C, C-6), 126.6 (1C, C-11), 131.7 (2C, C-12, C-16), 133.1 (1C, C-9), 136.0 (1C, C-4), 153.9 (1C, C-2), 159.6 (1C, C-14), 186.0 (1C, C-8) ppm); Anal. calcd for C_16_H_13_NO_2_: C, 76.48; H, 5.21; N, 5.57; found: C, 76.31 H, 5.15; N, 5.50 %; ESIMS (MeOH, *m/z*): 250 [*Z*-**1b** − H]^−^.

#### (Z)-2-(3,4-Dimethoxybenzylidene)indolin-3-one (Z-**1c**)

Orange crystals, yield 66% (211 mg, 0.75 mmol); mp 191–192 °С; *R*_f_ 0.56 (hexane/EtOAc 1:1, v/v); ^1^H NMR (500 MHz, DMSO-*d*_6_) δ 3.82 (s, 3H, H-18), 3.85 (s, 3H, H-17), 6.66 (s, 1H, H-10), 6.91 (t, *J* = 7.8 Hz, 1H, H-5), 7.06 (d, *J* = 8.4 Hz, 1H, H-15), 7.14 (d, *J* = 8.1 Hz, 1H, H-3), 7.29 (d, *J* = 2.0 Hz, 1H, H-12), 7.35 (dd, *J* = 8.7, 2.0 Hz, 1H, H-16), 7.51 (ddd, *J* = 8.3, 6.9, 1.1 Hz, 1H, H-4), 7.58 (d, *J* = 7.6 Hz, 1H, H-6), 9.66 (s, 1H, H-1) ppm; ^13^C NMR (126 MHz, DMSO-*d*_6_) δ 55.6 (1C, C-18), 55.7 (1C, C-17), 111.1 (1C, C-10), 112.0 (1C, C-15), 112.7 (1C, C-3), 113.8 (1C, C-12), 119.6 (1C, C-5), 120.4 (1C, C-7), 123.3 (1C, C-16), 123.9 (1C, C-6), 126.8 (1C, C-11), 133.3 (1C, C-9), 136.0 (1C, C-4), 148.9 (1C, C-14), 149.5 (1C, C-13), 154.0 (1C, C-2), 186.0 (1C, C-8) ppm; Anal. calcd for C_17_H_15_NO_3_: C, 72.58; H, 5.37; N, 4.98; found C, 72.80; H, 5.27; N, 4.89 %; ESIMS (MeOH, *m/z*): 280 [*Z*-**1c** − H]^−^.

## Supporting Information

File 1 Additional spectral data, detailed description of the experiments performed, NMR of compounds *Z*-**1a**–**c** and LED characteristics.
