# Supplementary material for: Design, synthesis and investigation of water-soluble hemi-indigo photoswitches for bioapplications
Source: Beilstein J Org Chem. 2019 Nov 22;15:2822–9. doi: 10.3762/bjoc.15.275 (PMC6880827; doi:10.3762/bjoc.15.275)
Supplement: File 1 — Additional spectral data, detailed description of the experiments performed, NMR of compounds Z-1a–c and LED characteristics. [file Beilstein_J_Org_Chem-15-2822-s001.pdf]

BEILSTEIN JOURNAL OF ORGANIC CHEMISTRY

Supporting Information

for

Design, synthesis and investigation of water-soluble hemi-indigo photoswitches for bioapplications

Daria V. Berdnikova

Beilstein J. Org. Chem. **2019,** *15,* 2822–2829. doi:10.3762/bjoc.15.275

Additional spectral data, detailed description of the experiments performed, NMR of compounds *Z*-1a–c and LED characteristics

License and Terms: This is a supporting information file under the terms of the Creative Commons Attribution License (http://creativecommons.org/licenses/by/4.0). Please note that the reuse, redistribution and reproduction in particular requires that the authors and source are credited.
The license is subject to the *Beilstein Journal of Organic Chemistry* terms and conditions: (https://www.beilstein-journals.org/bjoc)

Table of contents

1. Optical spectroscopy studies S2

1.1. Photoswitching of **1b** and **1c** in water at different irradiation wavelengths S2

1.2. Calculation of the extent of the *Z*–*E* conversion in PPS by the Fischer method S2

1.3. Calculation of the *E*-isomer half-lifes S3

1.4. Calculation of the photoisomerization quantum yields S5

2. NMR data S7

3. Characteristics of LEDs S13

4. References S13

1. Optical spectroscopy studies

*1.1. Photoswitching of **1b** and **1c** in water at different irradiation wavelengths*

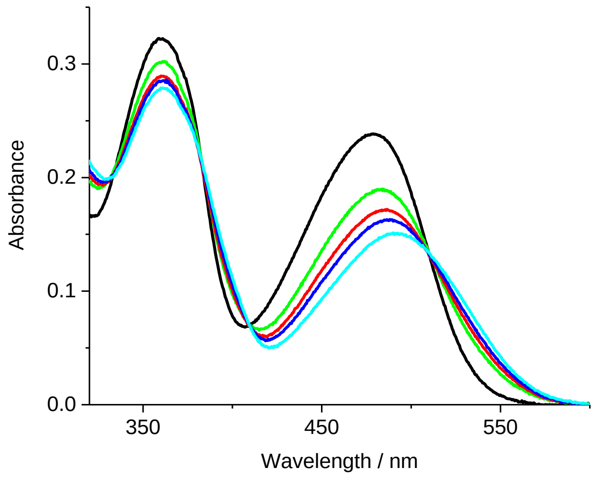

Figure S1: Absorption spectra of *Z*-**1b** (black) and photostationary mixtures of *Z*- and *E*-isomers of **1b** obtained upon irradiation with 360 nm (red), 420 nm (blue), 470 nm (cyan) and 520 nm (green) light in water with 2% EtOH, *c* = 20 μM.

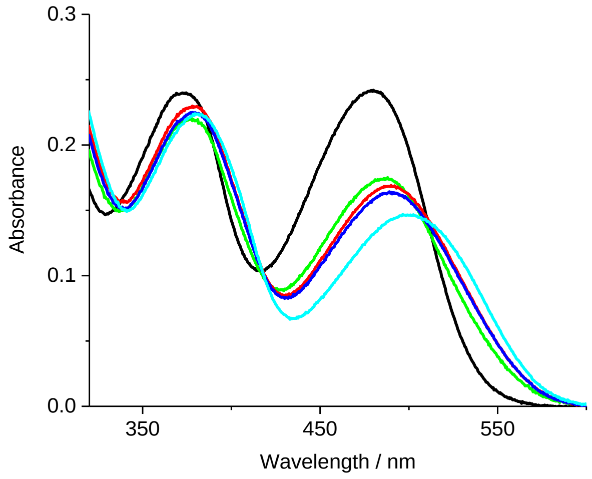

Figure S2: Absorption spectra of *Z*-**1c** (black) and photostationary mixtures of *Z*- and *E*-isomers of **1c** obtained upon irradiation with 360 nm (red), 420 nm (blue), 470 nm (cyan) and 520 nm (green) light in water with 2% EtOH, *c* = 20 μM.

1.2. Calculation of the extent of the Z-E conversion in PPS by Fischer method

The Fischer method was applied to determine the extent of the *Z*–*E* conversion α upon irradiation with 470 nm light and to calculate the absorption spectra of pure *E*-isomers of the hemi-indigo derivatives [1]. The Fischer method can be applied to reversible photochemical systems comprising only two interconverting components A ↔ B (starting form and photoinduced form) that are stable, both thermally and photochemically to make possible the establishment of true photostationary states. Thus, for every case the absorption spectra of the starting *Z*-isomers and of two photostationary states obtained by irradiation of the solutions of the corresponding *Z*-isomers at two different wavelengths (470 nm and 520 nm) were recorded (Figure 2, main text). Assuming that the ratio of the quantum yields of the backward and forward reactions ($\Phi_{Z\rightarrow E}/\Phi_{E\rightarrow Z}$) is independent of the irradiation wavelength, the fraction of the *Z*-isomer in each PSS, the absorption spectrum of the *Z*-isomer (Figure 2, main text) and the extent of *Z*–*E* conversion (Table 1, main text) were calculated. Based on the calculated absorption spectra of *E*-**1b** and *E*-**1c** and the experimental absorption spectra of *Z*-**1b** and *Z*-**1c**, the extents of *Z*–*E* conversion for PSS^{590} were calculated by the Fisher method, as described above (Table 1, main text).

1.3. Calculation of the E-isomer half-lifes

Thermal dark *E*–*Z* relaxation kinetics of hemi-indigo derivatives was followed by UV–vis spectroscopy at 60 °C. Considering that the thermal *E*–*Z* isomerization of the hemi-indigo scaffold is a unimolecular first order reaction that proceeds completely to the pure *Z*-isomer, Equation 1 can be applied [2]:

$$-\frac{d[E]}{dt} = \frac{d[Z]}{dt} = k[E] \qquad \text{(Eq. 1)}$$

where *k* is the rate constant of the thermal *E*–*Z* relaxation at a certain temperature.

The rate constants *k* were obtained from the monoexponential fitting of the thermal dark *E*–*Z* relaxation at 60 °C of the pre-irradiated by 470 nm light photostationary mixtures of *E*- and *Z*-isomers of **1b**, **1c** and **2** (Figure S3).

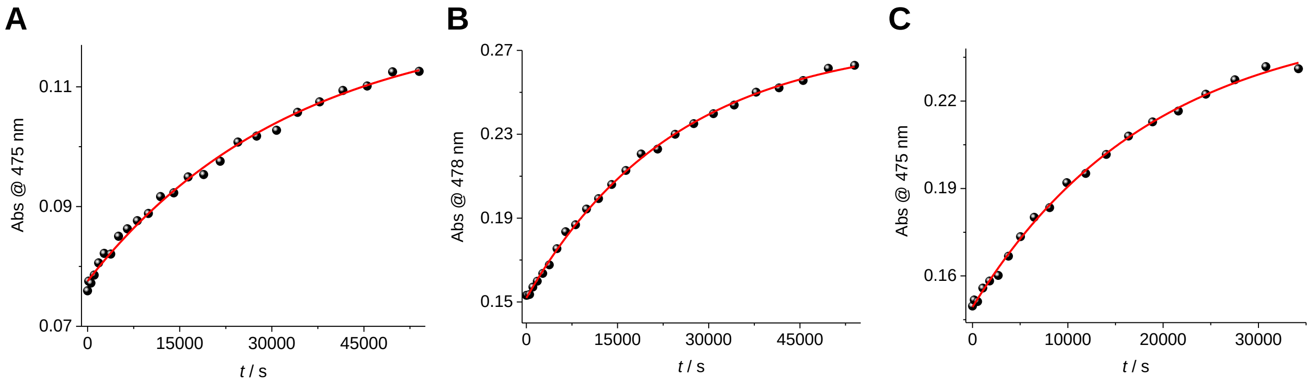

$k = (3.00 \pm 0.23) \times 10^{-5}\ s^{-1}$

$\chi^2 = 7.18131E\text{-}7$

$r^2 = 0.99506$

$k = (4.42 \pm 0.26) \times 10^{-5}\ s^{-1}$

$\chi^2 = 1.47066E\text{-}6$

$r^2 = 0.99839$

$k = (5.34 \pm 0.27) \times 10^{-5}\ s^{-1}$

$\chi^2 = 1.74731E\text{-}6$

$r^2 = 0.99794$

Figure S3: Evolution of the absorbance (black spheres) for the thermal *E*–*Z* isomerization in water at 60 °C of (A) **1b**, *c* = 10 μM, λ_{det} = 475 nm, solution contained 1% (v/v) EtOH for solubility reasons; (B) **1c**, *c* = 20 μM, λ_{det} = 478 nm; (C) **2**, *c* = 20 μM, λ_{det} = 475 nm. Solid red line indicates the fit of the experimental data to the monoexponential model.

The Eyring equation (2) was used for the calculation of the free activation enthalpy Δ*G** of the *E*–*Z* isomerization [2]:

$$k = \frac{k_B T}{h} e^{-\frac{\Delta G^*}{RT}} \qquad \text{(Eq. 2)}$$

where k_B is the Boltzmann constant, *h* is the Planck constant, *R* is the universal gas constant, *T* is temperature in K. Considering the numerical values of the constants, Equation 2 can be rearranged as following:

$$\Delta G^* = 8.314T \cdot \left(23.760 + \ln\left(\frac{T}{k}\right)\right) \qquad \text{(Eq. 3)}$$

Using the obtained Δ*G** values, the half-lifes of the *E*-isomer of hemi-indigo derivatives at 25 °C were calculated (Table 1, main text).

1.4. Calculation of the photoisomerization quantum yields

The quantum yield of *Z–E* isomerization was determined upon irradiation with 470 nm (blue) light. The quantum yield of *E–Z* isomerization was determined upon irradiation with 590 nm (amber) light. In the used setup, the irradiation was performed by placing a LED on the top of a photometric cell. The LED output went through the round aperture with a diameter of 3 mm. Reinecke salt $NH_4[Cr(NCS)_4(NH_3)_2]\times H_2O$ was used as a chemical actinometer for the determination of the intensity of the visible-light source [3]. A concentrated solution of Reinecke salt in water was prepared, filtered to remove undissolved solids and acidified to pH 5.3–5.5 by addition of a calculated amount of 0.01 M H_2SO_4. Samples of the prepared solution of the actinometer (V_{samp} = 0.35–0.40 mL) were irradiated for different time intervals; then aliquots (V_{aliq} = 0.05 mL) of the irradiated samples were taken and diluted with 6.2 mL of 0.1 M solution of $Fe(NO_3)_3$ in 0.5 M $HClO_4$ to the final volume (V_{dil} = 6.25 mL). The absorbance of the obtained mixtures was determined photometrically at the absorption maximum of iron(III) rhodanide (λ_{max} = 450 nm). The obtained absorbance values were plotted vs irradiation time and fitted to a linear dependence. The first derivative of the linear fit gave the *D/t* ratio.

The light intensity was calculated as follows:

$$I_0 = 0.001 \cdot \frac{V_{dil}}{V_{aliq}\, \varepsilon\, l} \cdot \frac{1}{\Phi} \cdot \frac{D}{t} \cdot \frac{1}{(1 - 10^{-\varepsilon' c' l})} \qquad \text{(Eq. 4)}$$

where I_0 is the intensity of the light source (einstein L^{-1} s^{-1}), Φ is the quantum yield of the rhodanide anion formation at the irradiation wavelength, ε is the extinction coefficient of iron(III) rhodanide at 450 nm (ε = 4300 M^{-1} cm^{-1}), and *l* is the irradiation path (cm).

The correction coefficient ($1 - 10^{-\varepsilon' c' l}$) was introduced to compensate the residual transmittance of the actinometer solution at the irradiation wavelength; ε′ is the extinction of the Reinecke salt at irradiation wavelength (ε'_{470} = 50.0 M^{-1} cm^{-1}, ε'_{590} = 38.9 M^{-1} cm^{-1}), *c′* is the actual concentration of the actinometer solution determined photometrically before the measurement (for irradiation experiments at 470 nm: *c′* = 0.0212 M; for irradiation experiments at 590 nm: *c′* = 0.0225 M). The obtained values of the light intensity are I_0^{470} = 3.841E−08 einstein L^{-1} s^{-1} for 470 nm LED and I_0^{590} = 1.088E−08 einstein L^{-1} s^{-1} for 590 nm LED.

Photoisomerization quantum yields were calculated according to Equation 5.

$$\Phi = \frac{\Delta A / \Delta t}{I_0 \left(1 - 10^{-A'}\right) \cdot \varepsilon \cdot 1000} \qquad \text{(Eq. 5)}$$

where $\Delta A/\Delta t$ is the change of absorbance at the detection wavelength within time, I_0 is the intensity of the light source, A' is the initial absorption of the sample at the irradiation wavelength, ε is the extinction coefficient at the detection wavelength.

For the forward *Z*–*E* switching of **1b**, **1c** and **2** that starts from 100% of the *Z*-isomer, $\Delta A/\Delta t$ corresponds to the initial slope of the *A* vs *t* kinetics recorded at the wavelength, where the absorbance of the *Z*-isomer is close to zero (λ_{det} = 565 nm for *Z*-**1b** and *Z*-**2**, λ_{det} = 560 nm for *Z*-**1c**). For the backward *E*–*Z* switching the initial solutions were pre-irradiated at 470 nm, therefore, the backward reaction started form the corresponding mixtures of *E*- and *Z*-isomers. To apply the initial slope method in this case, the *A* vs *t* kinetics were fitted to a polynomial dependence and extrapolated to the zero absorbance of the *Z*-isomer (Figure S4) [2]. The initial slope was obtained by the insertion of the extrapolated *t* value to the first derivative of the fitting polynomial.

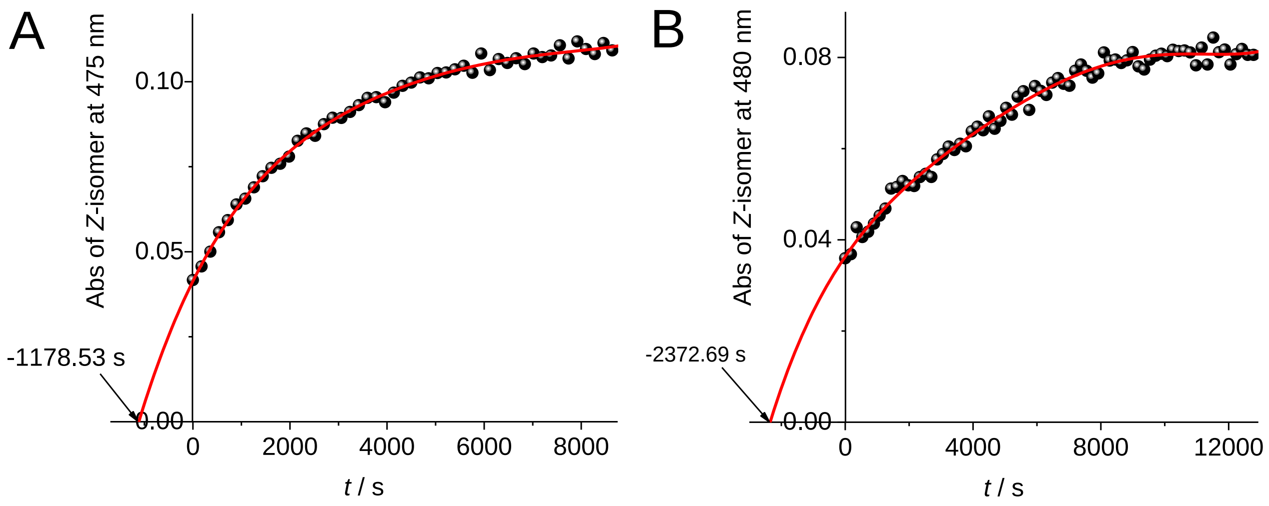

$y = 0.041 + 2.872\text{E-}5x - 6.371\text{E-}9x^2 + 9.375\text{E-}13x^3 - 8.021\text{E-}17x^4 + 2.882\text{E-}21x^5$	$y = 0.036 + 1.022\text{E-}5x - 1.544\text{E-}9x^2 + 2.408\text{E-}13x^3 - 2.100\text{E-}17x^4 + 6.564\text{E-}22x^5$
$y' = 2.872\text{E-}5 - 1.274\text{E-}8x + 2.812\text{E-}12x^2 - 3.208\text{E-}16x^3 + 1.441\text{E-}20x^4$	$y' = 1.022\text{E-}5 - 3.088\text{E-}9x + 7.224\text{E-}13x^2 - 8.004\text{E-}17x^3 - 3.282\text{E-}21x^4$

Figure S4: Quantum yield measurement of the *E*–*Z* photoisomerization of hemi-indigo derivatives (A) **1c** and (B) **2** upon irradiation with 590 nm starting from the pre-irradiated mixtures of *E*- and *Z*-isomers (PSS^{470}, black squares) in water, 20 °C. The whole photoconversion kinetics was fitted to the corresponding polynomials (red solid line).

2. NMR data

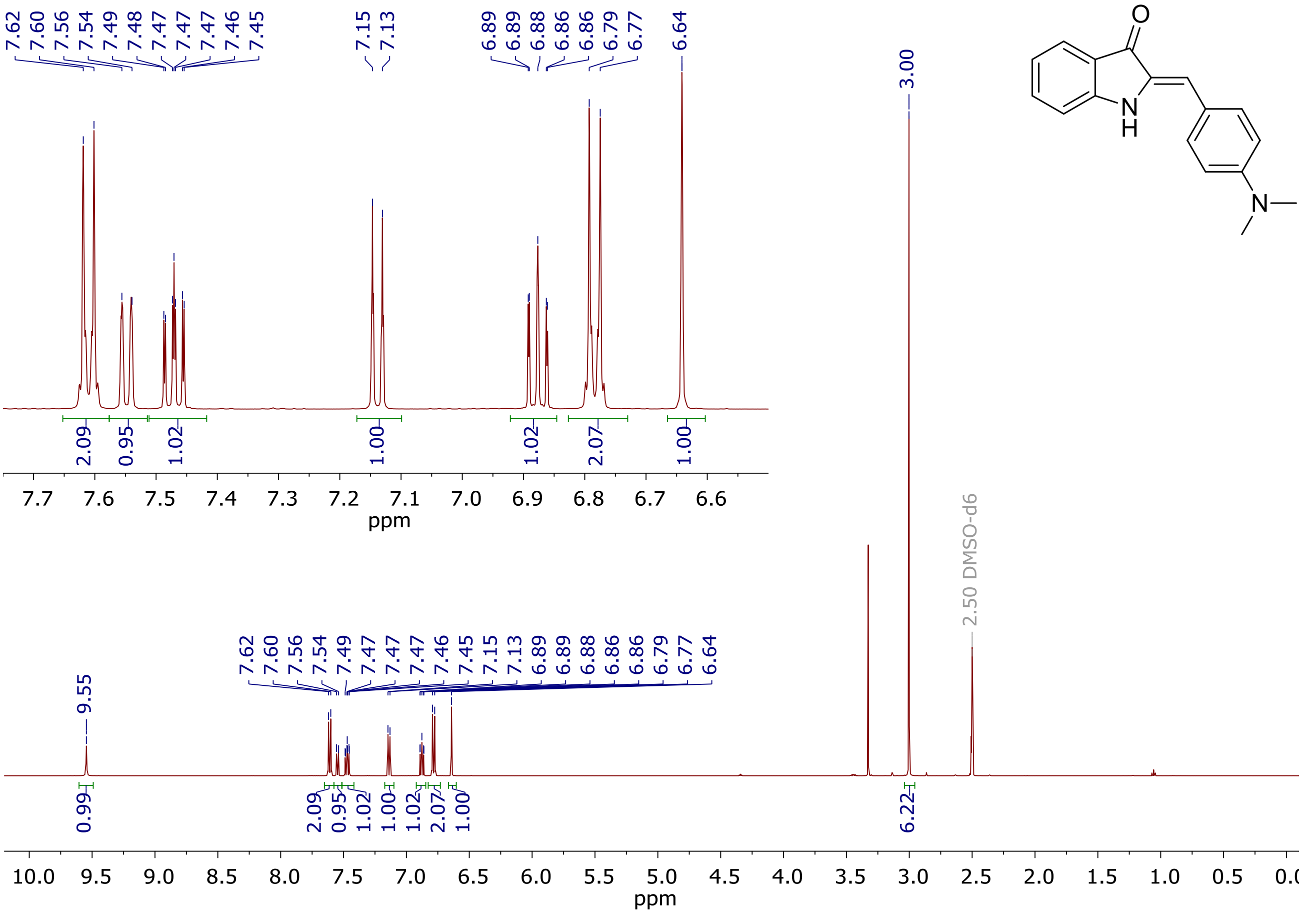

Figure S5: ^{1}H NMR spectrum of *Z*-**1a** in DMSO-d_6.

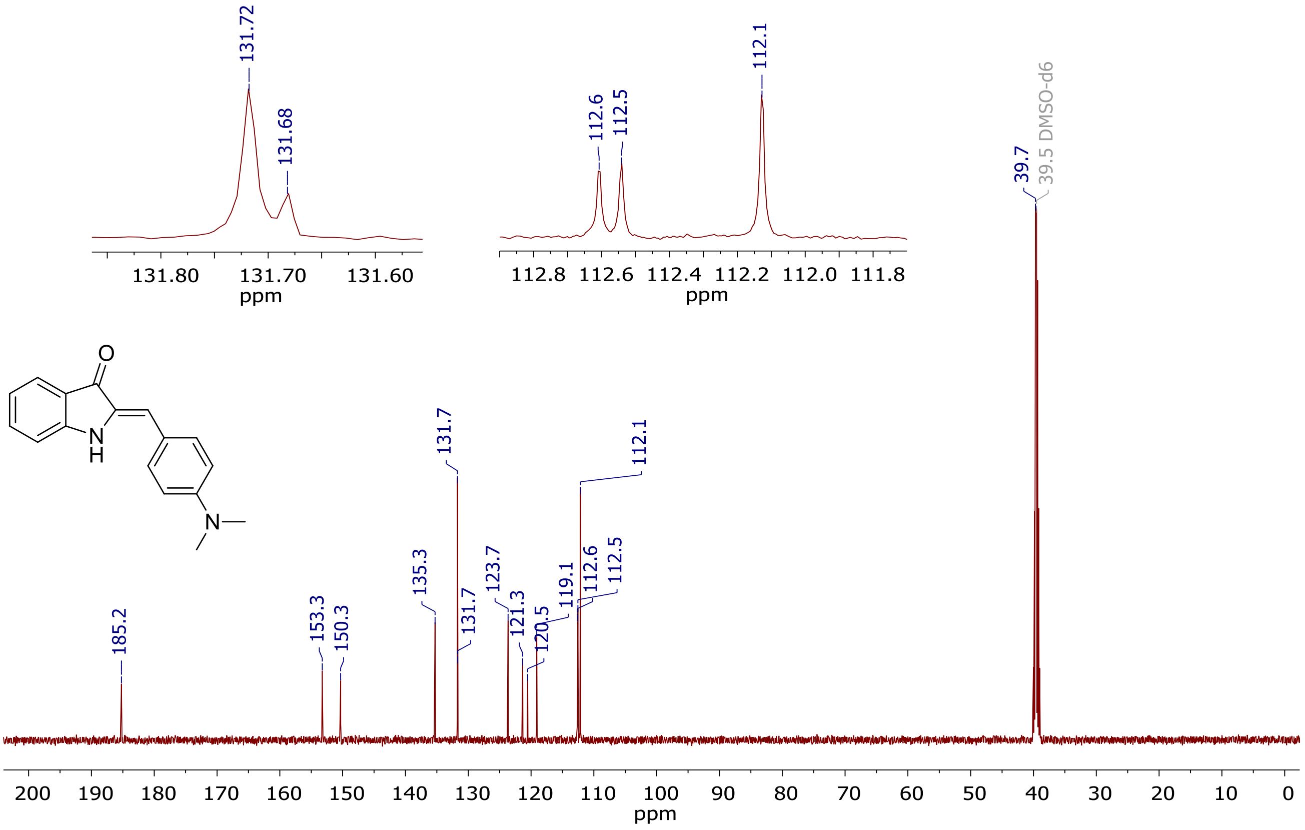

Figure S6: ^{13}C NMR spectrum of *Z*-**1a** in DMSO-d_6.

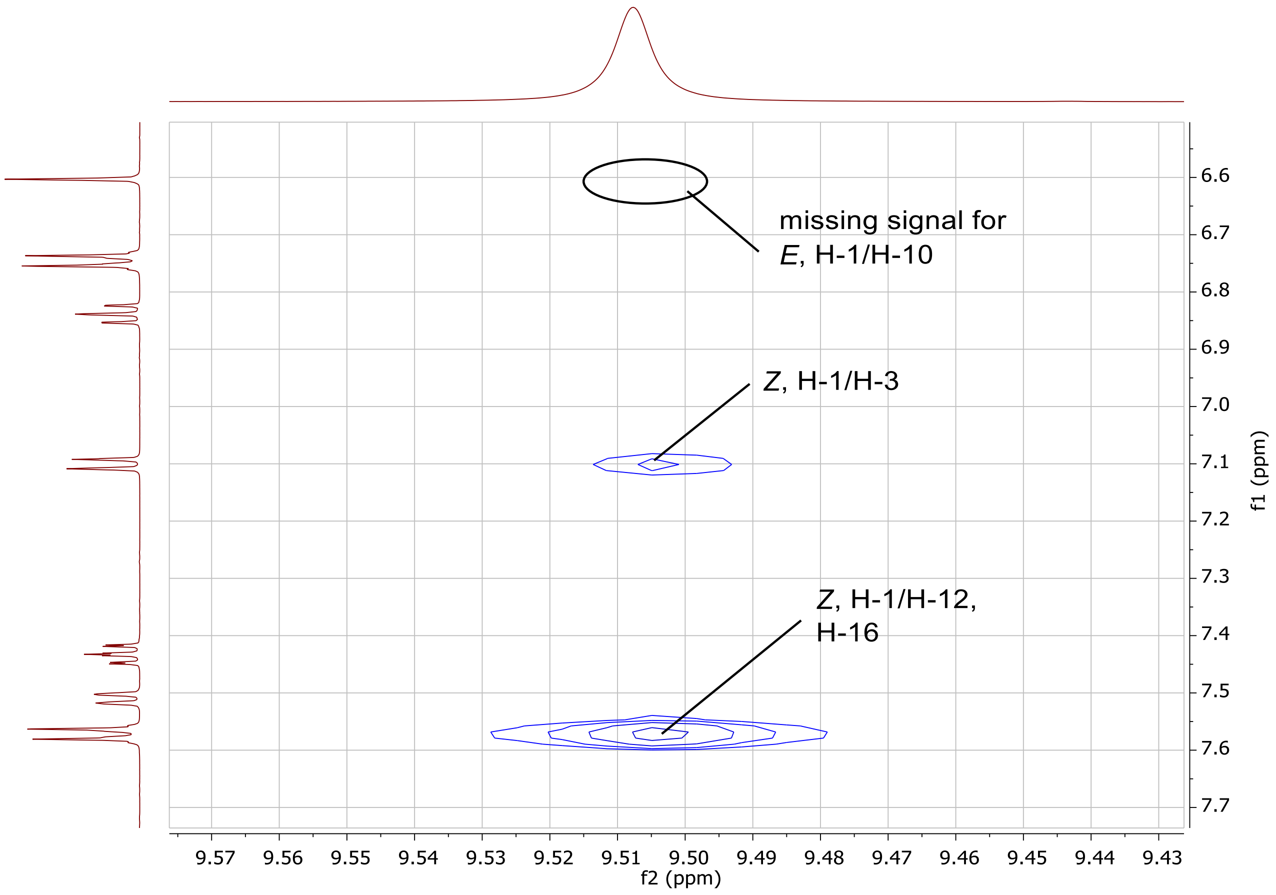

Figure S7: Section of the NOESY NMR spectrum of hemi-indigo *Z*-**1a** in DMSO-d_6. The NOE cross-signal between protons H-1 and H-12 indicates their close proximity and, therefore, proves the *Z*-conformation. In the *E*-conformation a cross signal between protons H-1 and H-10 is expected, but it is not observed.

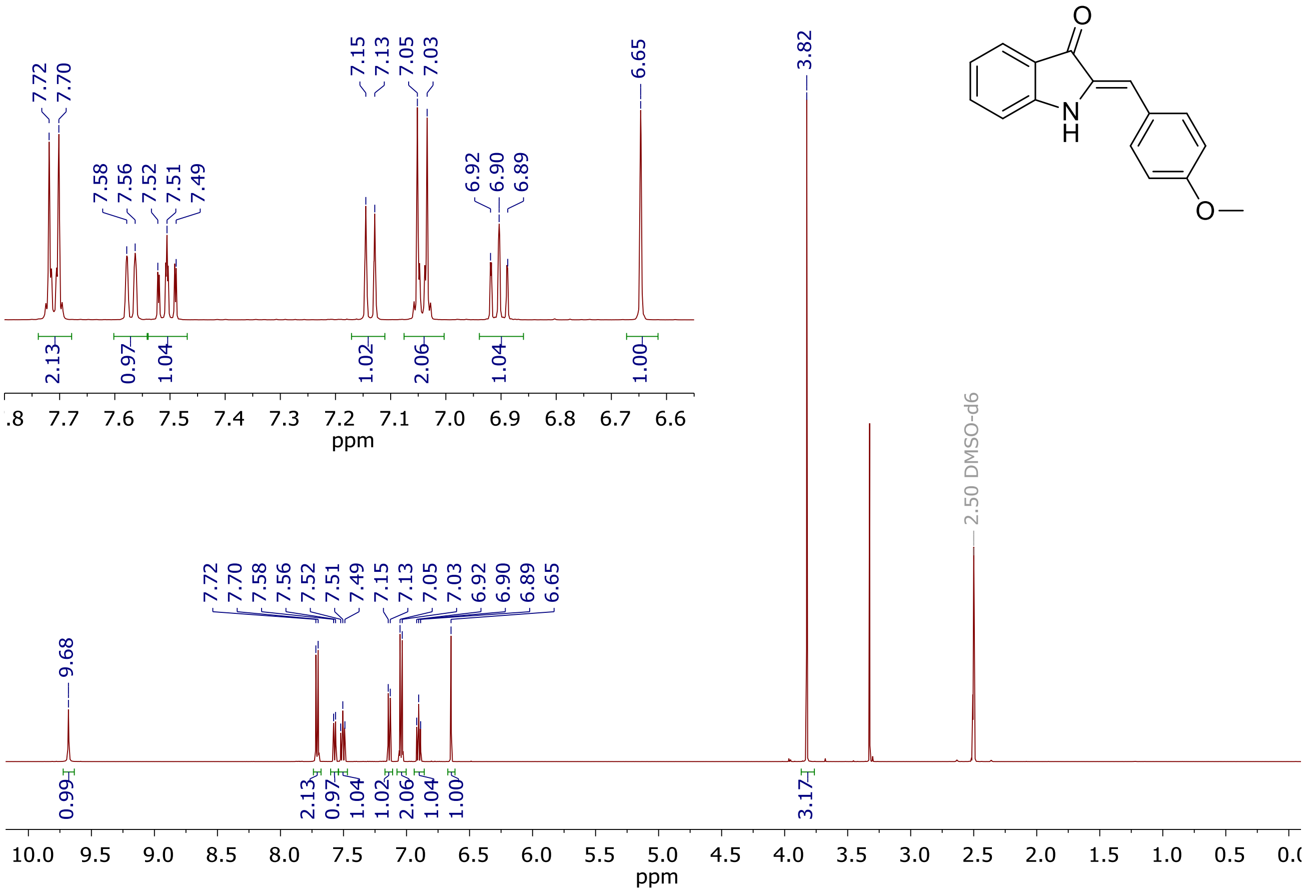

Figure S8: ^{1}H NMR spectrum of *Z*-**1b** in DMSO-d_6.

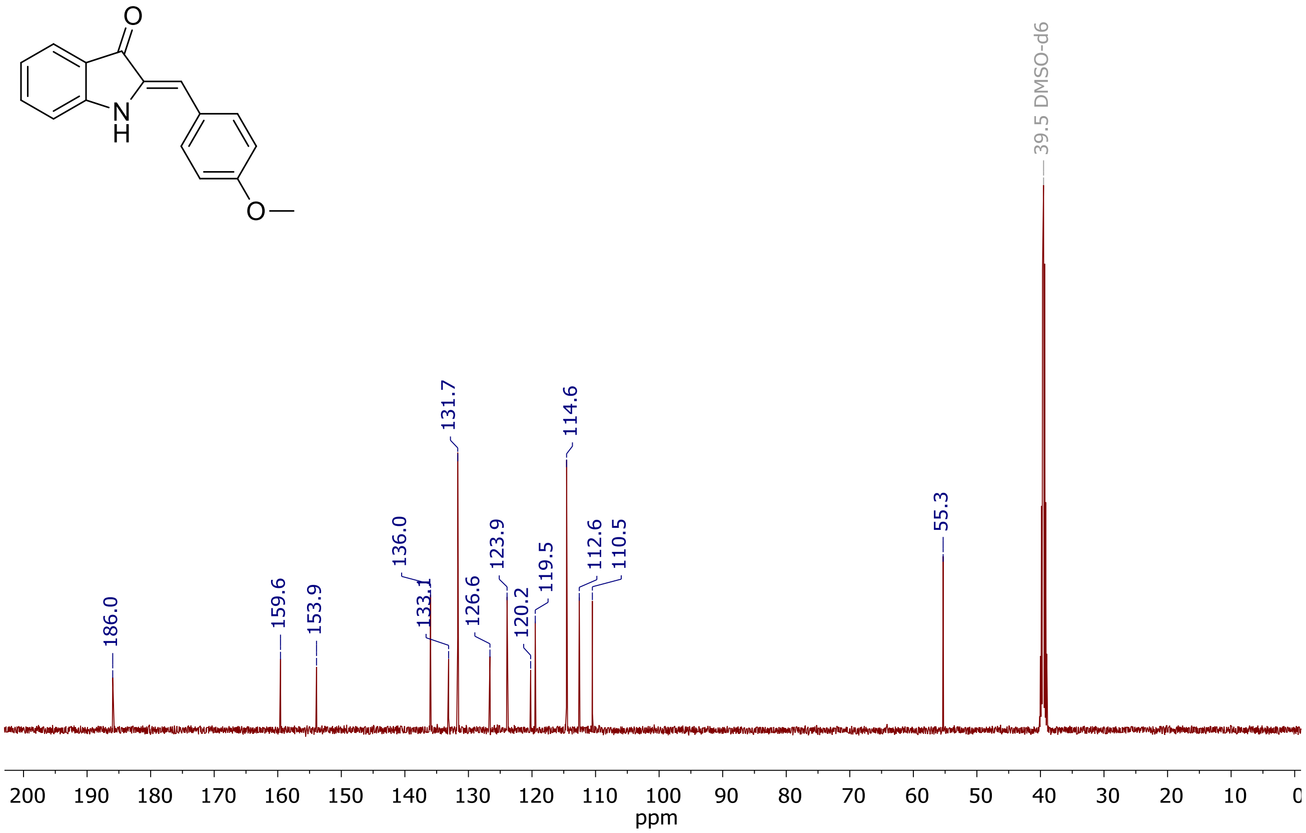

Figure S9: ^{13}C NMR spectrum of *Z*-**1b** in DMSO-d_6.

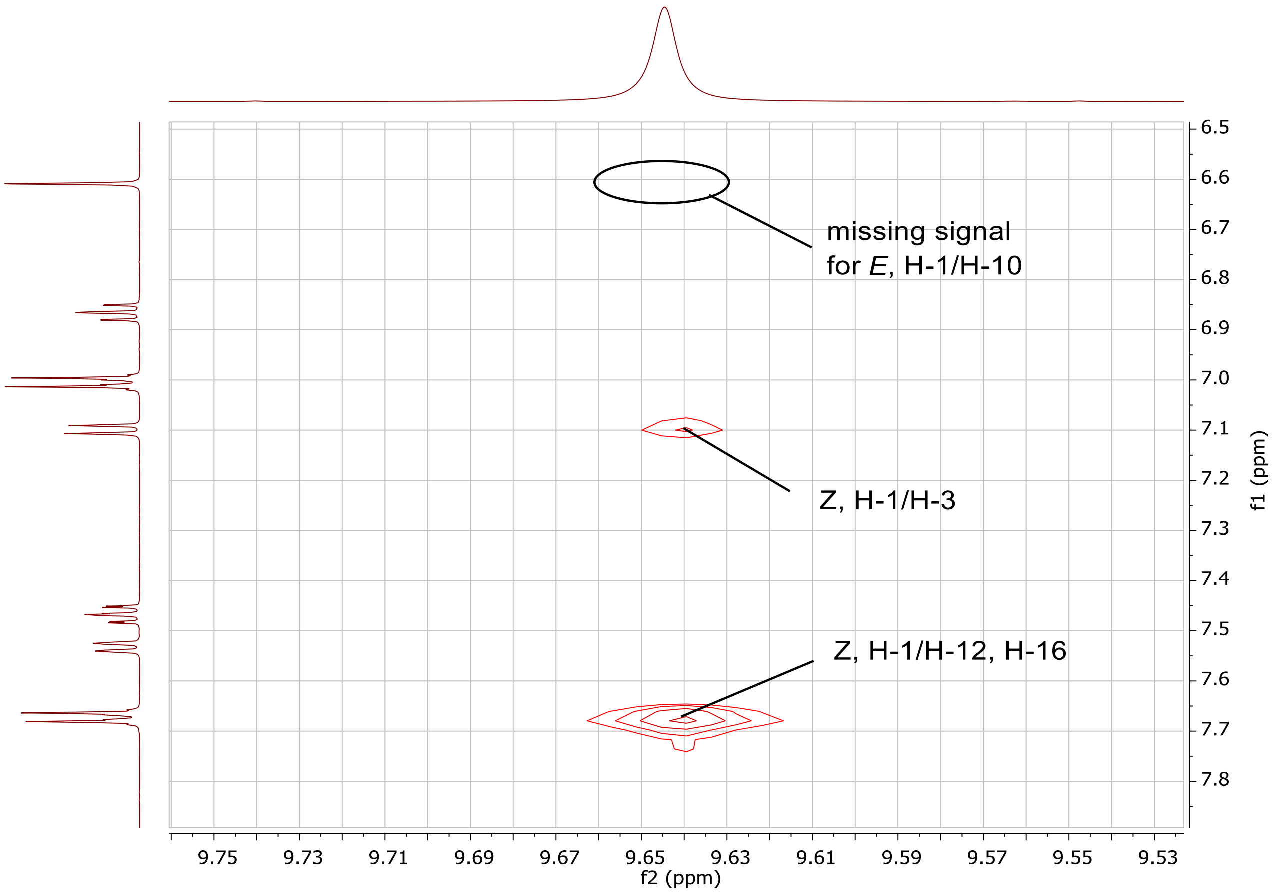

Figure S10: Section of the NOESY NMR spectrum of hemi-indigo *Z*-**1b** in DMSO-d_6. The NOE cross-signal between protons H-1 and H-12 indicates their close proximity and, therefore, proves the *Z*-conformation. In the *E*-conformation a cross signal between protons H-1 and H-10 is expected, but it is not observed.

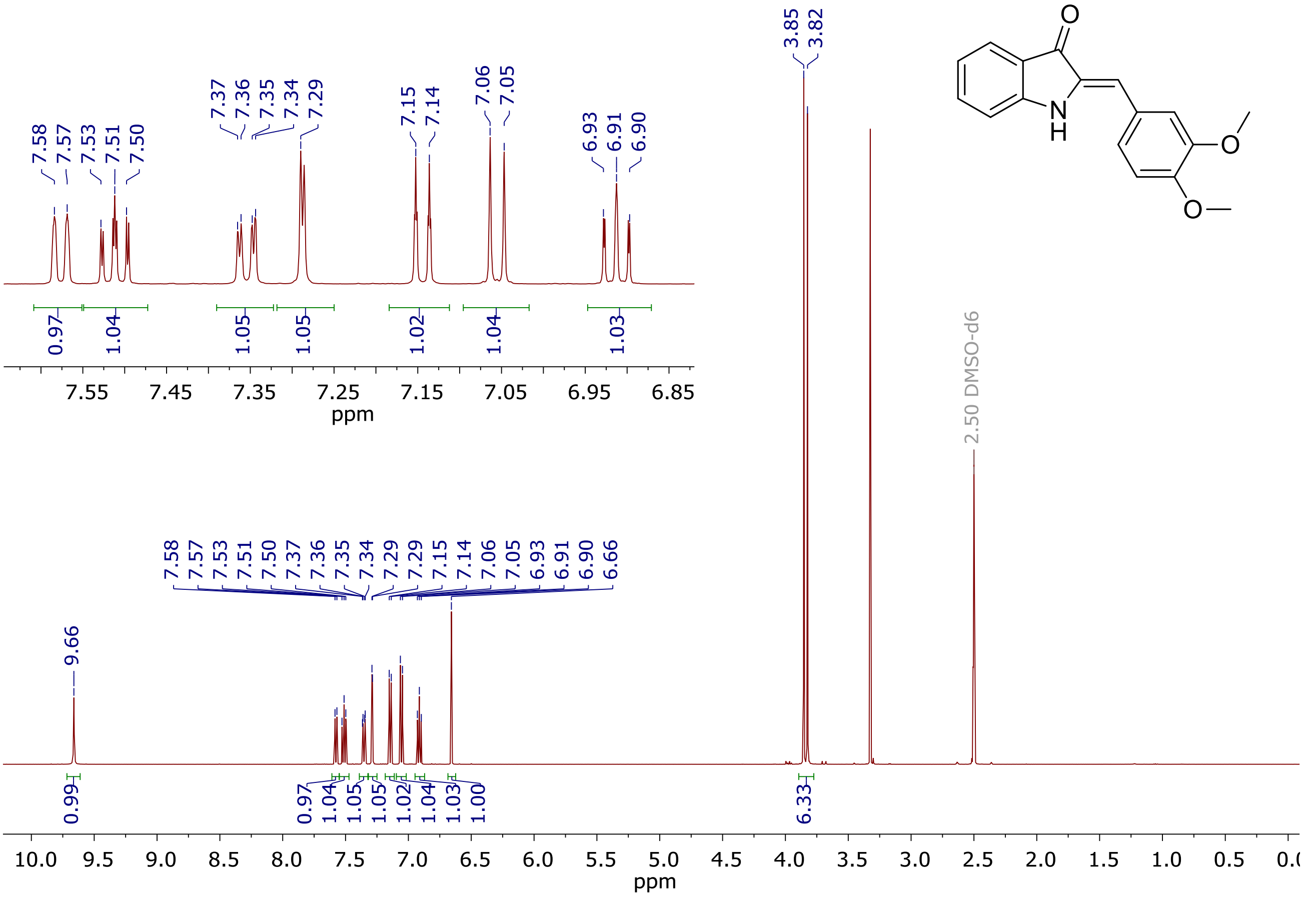

Figure S11: ^{1}H NMR spectrum of *Z*-**1c** in DMSO-d_6.

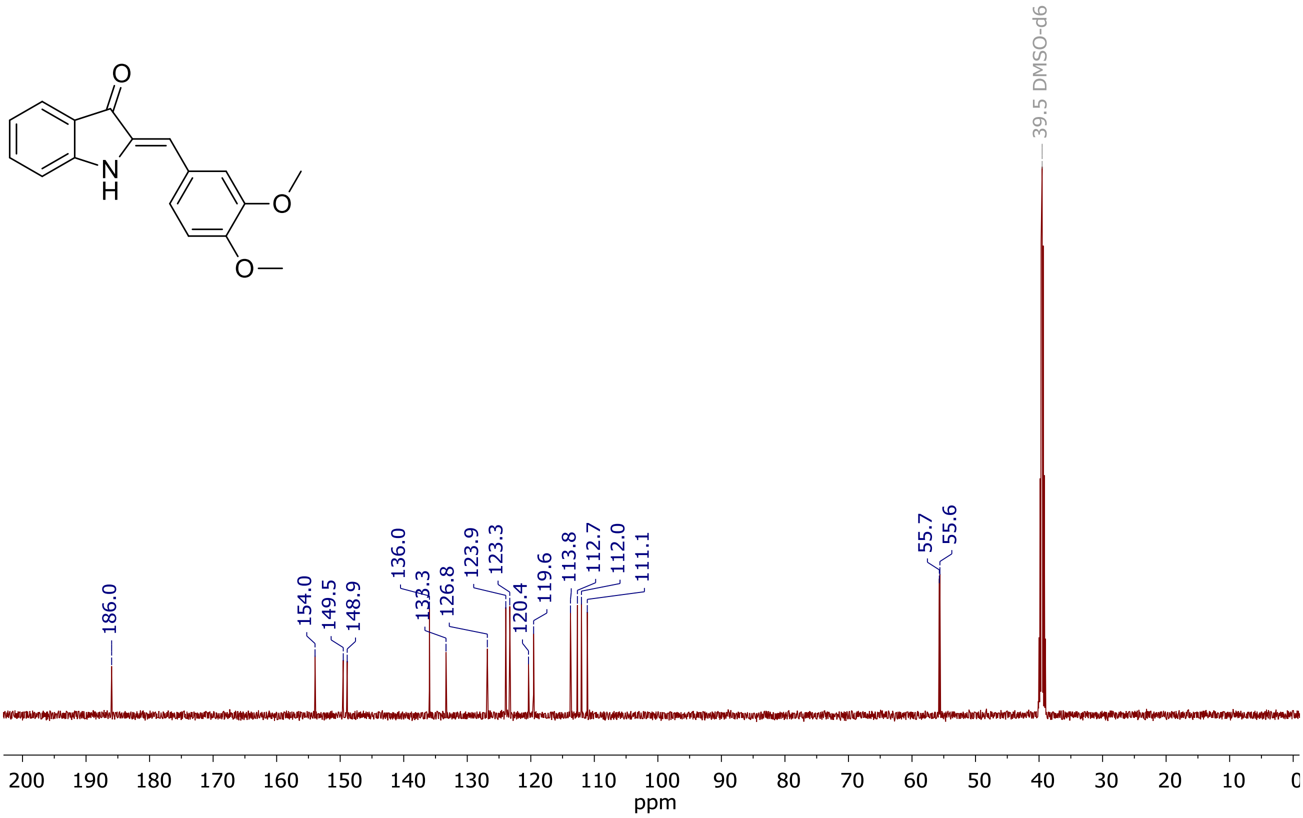

Figure S12: ^{13}C NMR spectrum of *Z*-**1c** in DMSO-d_6.

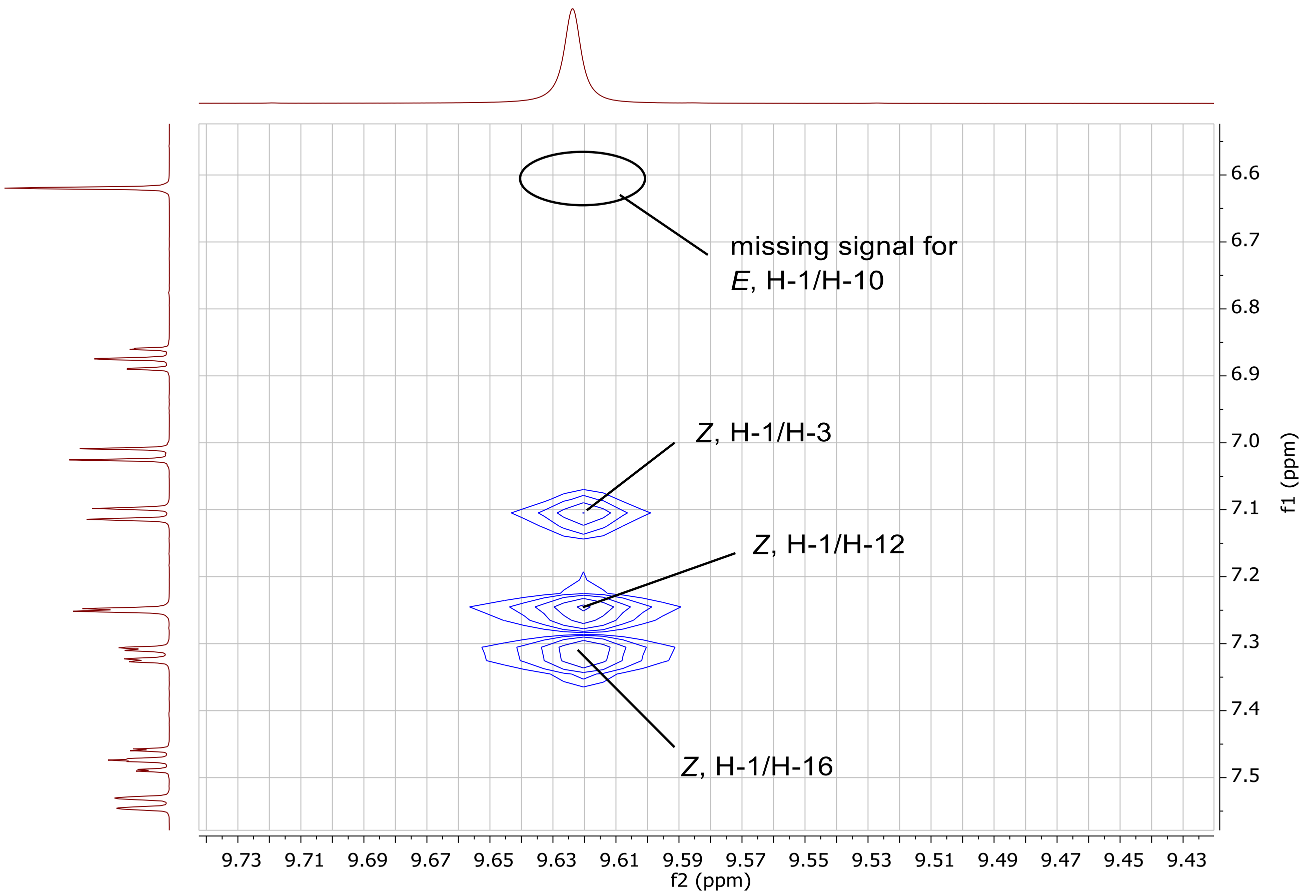

Figure S13: Section of the NOESY NMR spectrum of hemi-indigo *Z*-**1c** in DMSO-d_6. The NOE cross-signal between protons H-1 and H-12 indicates their close proximity and, therefore, proves the *Z*-conformation. In the *E*-conformation a cross signal between protons H-1 and H-10 is expected but it is not observed.

3. Characteristics of LEDs

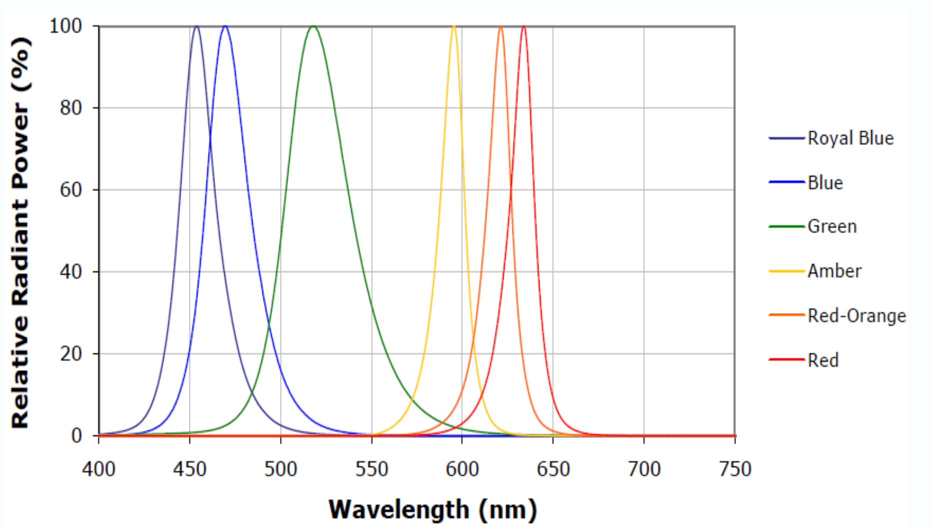

Figure S14: Emission of the visible-range LEDs (spectra are taken from the producer's specification). In this study, blue (470 nm), green (520 nm) and amber (590 nm) LEDs were used.

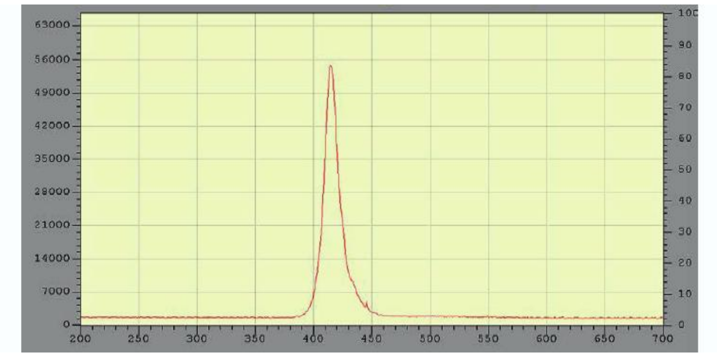

Figure S15: Emission of the blue LED (420 nm) (spectrum is taken from the producer's specification).

4. References

[1] E. Fischer, *J. Phys. Chem*. **1967**, *71*, 3704.

[2] C. Petermayer, S. Thumser, F. Kink, P. Mayer, H. Dube, *J. Am. Chem. Soc*. **2017**, *139*, 15060.

[3] E. W. Wegner, A.W. Adamson, *J. Am. Chem. Soc*. **1966**, *88*, 394.
